# Delays in seeking and receiving health care services for pneumonia in children under five in the Peruvian Amazon: a mixed-methods study on caregivers’ perceptions

**DOI:** 10.1186/s12913-018-2950-z

**Published:** 2018-03-01

**Authors:** Mónica J. Pajuelo, Cynthia Anticona Huaynate, Malena Correa, Holger Mayta Malpartida, Cesar Ramal Asayag, Juan R. Seminario, Robert H. Gilman, Laura Murphy, Richard A. Oberhelman, Valerie A. Paz-Soldan

**Affiliations:** 1 Office of Global Health, Tulane University School of Public Health and Tropical Medicine, 1140 Canal Street, Suite 2210, New Orleans, LA 70112 USA; 2 Department of Cellular and Molecular Science. School of Science and Philosophy, Universidad Peruana Cayetano Heredia, Lima, Peru; 3 Hospital Regional de Loreto, Iquitos, Peru; 4 Universidad Nacional de la Amazonia Peruana, Iquitos, Peru; 5 Department of International Health, Johns Hopkins Bloomberg School of Public Health, Baltimore, MD USA; 6 Department of Global Community Health & Behavioral Sciences, Tulane University School of Public Health and Tropical Medicine, New Orleans, LA USA

**Keywords:** Child pneumonia, Delays, Seek care, Caregiver, Health system

## Abstract

**Background:**

Delays in receiving adequate care for children suffering from pneumonia can be life threatening and have been described associated with parents’ limited education and their difficulties in recognizing the severity of the illness. The “three delays” was a model originally proposed to describe the most common determinants of maternal mortality, but has been adapted to describe delays in the health seeking process for caregivers of children under five. This study aims to explore the caregivers’ perceived barriers for seeking and receiving health care services in children under five years old admitted to a referral hospital for community-acquired pneumonia in the Peruvian Amazon Region using the three-delays model framework.

**Methods:**

There were two parts to this mixed-method, cross-sectional, hospital-based study. First, medical charts of 61 children (1 to 60 months old) admitted for pneumonia were reviewed, and clinical characteristics were noted. Second, to examine health care-seeking decisions and actions, as well as associated delays in the process of obtaining health care services, we interviewed 10 of the children’s caregivers.

**Results:**

Half of the children in our study were 9 months old or less. Main reasons for seeking care at the hospital were cough (93%) and fever (92%). Difficulty breathing and fast breathing were also reported in more than 60% of cases. In the interviews, caregivers reported delays of 1 to 14 days to go to the closest health facility. Factors perceived as causes for delays in deciding to seek care were apparent lack of skills to recognize signs and symptoms and of confidence in the health system, and practicing self-medication. No delays in reaching a health facility were reported. Once the caregivers reached a health facility, they perceived lack of competence of medical staff and inadequate treatment provided by the primary care physicians.

**Conclusion:**

According to caregivers, the main delays to get health care services for pneumonia among young children were identified in the initial decision of caregivers to seek healthcare and in the health system to provide it. Specific interventions targeted to main barriers may be useful for reducing delays in providing appropriate health care for children with pneumonia.

## Background

Worldwide, pneumonia continues to be the leading cause of child mortality, causing about 1.2 million deaths in children under-five annually [1–3] about 95% of deaths are disproportionally found in low and middle income countries [4]^.^. About 80% of these deaths occur during the first 2 years of life [2, 5].

In Peru, deaths due to acute respiratory infections, including pneumonia are the third leading cause of under 5 child mortality, after deaths related to prematurity and congenital anomalies [6]. Prompt care seeking and adequate management [7, 8] have been consistently recommended as key factors to reduce the global burden of child mortality due to pneumonia. Seeking timely care in a formal health facility is very important; however, for this to occur, parents or caregivers need to be able to identify the warning symptoms and signs of pneumonia. In the past decade, Peruvian public health efforts have focused on pneumonia prevention--specifically, through conjugated pneumococcal and Hib vaccines implementation--and on educational and awareness campaigns targeting vulnerable populations (i.e., increasing awareness about emergency signals when facing respiratory illness or distress). Despite these efforts, pneumonia mortality rates remain unchanged (1.2 deaths per 100 pneumonia episodes) among children under 5 years of age, being highest in the Highlands and in the Amazon rainforest [9].

To develop strategies to reduce child mortality due to pneumonia, it is necessary to understand the underlying factors contributing to morbidity and mortality. It is difficult to obtain population-based morbidity and mortality data from countries most affected by pneumonia [2]; hence, some studies have used verbal autopsies [1, 10], others have tried to examine this topic in clinical settings [5, 11]. A number of risk factors for pneumonia mortality have been identified, including a child’s young age, lack of exclusive breastfeeding, under-nutrition, congenital conditions [2, 5, 12–15], hypoxia, incomplete immunizations, immunodeficiency conditions, and different etiologic agents [12, 14, 15].

Previous studies have also examined the role of delays in receiving adequate care in mortality from pneumonia. Three studies in rural settings, one in Asia and two in Africa, found several factors that delayed receiving prompt attention at health care facilities [5, 11, 16]. Other studies in Africa described delays in seeking care, associated with parents’ limited education and not recognizing the severity of the illness [5, 17, 18], their use of traditional treatments [16, 18], self-medication of antibiotics at home [17, 18]. In Peru, one study based on junior physicians perceptions reported that health care seeking in rural areas was delayed because caregivers prioritize other activities–mainly economic and/or of subsistence over health care seeking [19]. Another study in the Andean highlands of southern Peru (Puno) investigated the social determinants of child mortality due to pneumonia and reported that delays in seeking care, flaws in health care delivery, payments and self-medication were contributing factors for the incidence and mortality rates due to pneumonia. [20].

Despite the described findings, there is a need to examine the factors associated to seeking and receiving care to manage child pneumonia in Peru, especially because of the great diversity among several regions in terms of geographical, climate and social factors, which may condition a change in the attitudes and practices of both caregivers and healthcare providers, and because there is lack of evidence/studies from the Peruvian Amazon Region. Moreover, Loreto showed in the past years previous to this study the highest levels of community-acquired pneumonia (CAP) and death due to pneumonia in children under 5 years old, as reported by the Ministry of Health. The objective of this study was to explore the caregivers’ perceived factors associated to seeking and receiving prompt health care services in a group of children hospitalized for CAP in a hospital of Iquitos, the capital city of Loreto, Peru, in the Amazonian lowlands. As a secondary objective, we investigated the clinical characteristics of a broader group of children admitted for CAP in the same hospital.

**Fig. 1:**
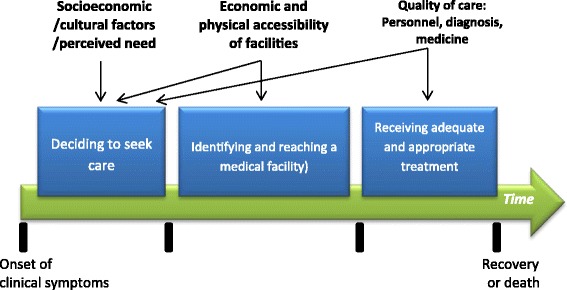
Three-Delays model and factors associated adapted from Thaddeus and Maine. Phases of the three delays for child pneumonia in a time framework

The “three delays model” was originally proposed to describe the most common determinants of maternal mortality [21]. It has been adapted to explore factors influencing care-seeking behaviors. This model describes the delays that could result in a bad outcome if not receiving prompt health care: the first delay being deciding to seek care, the second delay being identifying and reaching a health facility, and the third delay in receiving health care in a timely manner once in a health facility (Fig. 1). One recent comprehensive study conducted in rural communities in India [18] applied an adapted “three-delays model”, in structuring interviews and focus groups with caregivers of children under five, focusing on early identification of warning signs and knowledge of the health care seeking process (or lack thereof). We used this model as a framework for our study.

## Methods

### Study site

The Department of Loreto is located in the northern area of the Amazon rainforest in Peru. It has a population of 891,732, with approximately 55% of the population living in the district of Maynas, where the city of Iquitos is located [22]. Loreto, with a total area of 368,851.95 km^2^, is the largest of Peru’s 24 departments, encompassing over a quarter of the total area of the country. Loreto has a dispersed population (second lowest population density of the 24 departments); most communities are located on either side of the five main rivers of the region.

In terms of primary health care facilities, almost every community in Peru (rural and urban) has access to a health post aimed to provide promotion and prevention services and to manage other diseases including child diarrhea and pneumonia by using algorithms and basic medicines. Health posts do not have many diagnostic tools (tests and equipment) and most health posts in rural areas are in charge of junior medical doctors lacking experience. Therefore complicated cases should be referred to other health facilities located in the capital of each province or tertiary hospitals located in the cities.

This study was based in the Hospital Regional de Loreto (HRL), the main regional hospital located in Iquitos. It is a referral hospital for patients from all of Loreto, but it mostly receives patients from the urban area.

In past years, there has been a steady increase in the child mortality rate due to pneumonia in Loreto (from 1.3 in 2010 to 2.4 in 2013) [9]. In 2013, the Regional Directorate of Health of Loreto reported 63 deaths, with 55% reported in hospitals [23].

### Study design

This was a mixed-methods cross-sectional study, conducted between February and November 2014. Initially, we reviewed clinical chart data from children aged 1-60 months admitted with pneumonia to the HRL, in order to identify and recruit potential participants. This led to the main objective of this study which was to conduct semi-structured interviews (SSI) on a subsample of caregivers of the children admitted to the HRL with pneumonia.

As our primary goal was to get insights into the caregivers’ perceived factors associated to seeking and receiving prompt adequate care in children hospitalized for CAP, we aimed to do at least 30 interviews or until we got saturation. In the previous years an average of 100 children were hospitalized for CAP in a 12-months period. For the review of children’s medical charts, whenever a child was hospitalized, the research assistant (nurse) verified the diagnosis of community acquired pneumonia CAP, based on both the diagnosis at the emergency room, and confirmed with the diagnosis of the pediatrician at the pediatric ward. CAP was defined as the infection, as stated by the physicians, that has been acquired in the community (outside of the hospital). She then contacted the mother, father or legal authorized representative (LAR) to describe our study to them and ask for her or his authorization to review the child’s medical chart. If the father, mother or LAR gave informed consent, we reviewed the medical chart. We used an adapted version of the medical chart review developed by Kalter [24]. The following data was collected from medical charts: age, sex, reasons for seeking care at the hospital, vaccination history, and evaluation at admission, diagnosis, and treatment. Children were excluded from the final analysis if they had known AIDS or if they had any other condition known to cause severe immune -, which would put them at higher risk of dying in the event of any infection independently of other factors.

Participants of semi-structured interviews (SSI) were recruited from the pediatric ward during the child’s stay at the HRL. Caregivers were initially contacted by the research assistant who described this part of study to them. Then, if the caregivers were interested in participating, the field worker contacted them within a month to set up a time to meet. After reading and signing the informed consent, interviews took place at the caregivers’ home or, in some cases, in a private space in the hospital.

The SSI guide used for caregivers was a translated version of the questionnaire developed by Kalter [24] to explore care-seeking actions. Specifically, in 2004, Kalter developed a series of tools for caregivers of ill children, which focus on identifying the main causes of child’s death and include a “pathway analysis chart” to track the pathway to survival, including the assessment of care-seeking behaviors [24]. Briefly, it includes questions that are intended to explore step-by-step what caregiver did since the first appearance of symptoms (Table 1).

**Table 1:** Sections of the SSI guide

Caregivers’ Background	­ Relationship to child ­ Educational level ­ Occupation ­ Number of children ­ Socioeconomic indicators such as language(s) spoken, whether the family had access to piped water, type of floor, number of rooms, number of family members living in the same house ­ Distance to the closest health facility
Background of the child	­ Age ­ Sex ­ Breastfeeding history
Open ended question regarding pneumonia event	­ An open ended question, with no interruption or prompting, regarding the caregivers’ experience identifying and managing the pneumonia event
Pathway analysis chart	­ The pathway analysis chart involves systematic questions about the chronological actions that the caregiver took as soon as she/he realized that the child was sick: Who the caregiver asked for help; what this person did or told her/him to do; did the caregiver take the child to a health provider or not; what were the reasons for taking the child (or not); how many days lapsed between actions.

After reviewing the questionnaire, the research team translated the questions of the selected sections. There were no changes in the wording, since the original instrument was also developed in Peru. The questionnaire was then reviewed by the field team in Iquitos who piloted the questions as part of the field workers’ training.

All interviews were conducted in pairs: one of two investigators paired up with one of two local field workers. The two field workers were Red Cross collaborators who perform a range of activities, including health promotion, prevention campaigns and research in the rural and urban communities in Loreto. The field workers were sensitive and attuned to the population’s cultural customs and beliefs, the local Spanish dialect, as well as their health-related practices. They received a 2-day training by the investigators to conduct the interview. Because of the possibility that the field workers might interview caregivers whose children had died, the team also received training from a Red Cross specialist in dealing with psychological stress and trauma. The SSI guide had four sections. Table 1 describes the sections and information covered.

The last two sections of the SSI guide were designed to provide a comprehensive assessment of the decisions and actions taken by the caregivers from the onset of the symptoms to the admission at the HRL.

### Analysis

#### Data from the semi-structured interviews

All interviews were transcribed. Codes were developed based on the main themes explored in this study, as well as other emergent themes. The research team established a priori codes based on the three-delays model, and emergent sub-codes were added based on the topics identified in the transcripts. A codebook was developed and the research team returned to previous transcripts to ensure that codes were applied accordingly. Each transcript was manually coded by two investigators. Differences in coding were then discussed until agreement was reached.

#### Data from the medical charts

Data collected from the participants’ children’s medical charts were entered in Access © (2010 Microsoft Corporation), and descriptive statistics were conducted using Access and STATA12 (Stata Corporation, College Station, TX, USA). Means, medians and proportions are presented for variables of interest, and 95% CI were estimated with standard methods.

#### Ethical considerations

This study was approved by Institutional Review Boards at Tulane University (protocol # 514917–8), Universidad Peruana Cayetano Heredia (Protocol # 62073), and approval to conduct the study was also obtained from the Hospital Regional de Loreto (Memo # 2136, 12/05/2013). Written informed consent to review children’s medical charts was obtained from parents, as well as for interviews with caregivers.

## Results

### Review of Clinical Charts

From February to November our nurse contacted 69 children who were confirmed to have pneumonia at the entry at the pediatric ward. Out of them, sixty three parents consented to having their children’s medical charts reviewed; however, two were excluded due to other health complications that were the likely underlying cause of child hospitalization and mortality. One child was suspected of having AIDS; the other child seemed to be suffering from a severe neurological disease. Hence, we report our findings from the review of 61 clinical charts.

### Clinical characteristics of children admitted with pneumonia to HRL

Children were between 1 to 60 months old; 35 (56.5%) were boys and 26 (43.6%) were girls. Median age was 9 months old (IQR: 4–17). Three of the participants died due to CAP: two were boys (both 3 months old) and one was a girl (one month old). Table 2 shows the clinical characteristics of children admitted with CAP to HRL.

**Table 2:** Clinical characteristics of children admitted with CAP to HRL

Variable	*N*	(%)
Age, in months (median (IQR))^a^	9	4–17
Sex (Girls)	26	43.6%
Reasons for seeking help at the hospital		
Cough	57	93.4%
Fever	56	91.8%
Difficulty breathing	40	65.6%
Fast breathing	37	61.7%
Symptoms duration (median days (IQR))^a^	6	4–7
Symptoms at admission		
Cough	45	73.8%
Fast breathing (*n* = 58)^b^	42	72.4%
Fever	26	42.6%
Intercostal retraction	38	62.3%
Subcostal retraction	22	36.7%
Hemoglobin (median (IQR)^a^	10.2	9.3–11.0
Percentage of oxygen saturation (*n* = 33)(median (IQR))^a^	93	90–96
Oxygen administration	37	62.7%
Hospitalization days (median (IQR))^a^	5	4–7
Previous History		
Previous pneumonia	11	18.3%
otitis	0	
Vaccines administered at birth		
BCG	52	86.7%
Hepatitis B	45	79.0%

^a^(median (IQR)): In these rows, values shown under (*n*) are median values and values shown under the % column are interquartile ranges

^b^Fast breathing was defined as follows: If child was less than 2 months old: 60 breaths per minute (bpm) or more, if child was 2–12 months old: 50 bpm or more, or if child was 1–5 years old: 40 bpm or more [47]

The two main reasons participants gave for seeking care at the hospital for their children was because of cough (93.4%) and fever (91.8%). This was followed with difficulty breathing (65.6%) and fast breathing (61.7%); in fact, the three cases of infants who died were taken there mainly because of fast breathing or difficulty breathing. The median reported time for symptoms duration before going to the hospital was 6 days (IQR 4–7); however, this may be an underestimate because during our SSI, we compared what we found in the clinical report with what our interviews explored in depth with the participants, and we found differences. At the hospital they usually reported less time than what we found during the SSI.

At first evaluation after admission, physicians reported that most of the children had cough (73.8%) and fast breathing (72.1%); other important symptoms included intercostal (62.3%) and subcostal (36.7%) retractions. Measures of oxygen saturation were reported in only 33 children; the oximeter is not always available and hence oxygen saturation was not always measured. The median value was 92.4 (IQR 90%–96%). Oxygen saturation values reported for children who died were 85% in one child and 90% in the other (oxygen saturation was not evaluated in the third). Median hemoglobin was 10.2 (9.3–11), being lower in two children who died (8, and10; we do not have data of the third child). Also, in the clinical charts, providers reported a subjective observation of nutritional “appearance”, from overweight, normal, thin, to very thin. Up to 66% of children brought in were categorized as thin or very thin, and a third were categorized as having a normal appearance. Regarding vaccinations that children should have received at birth, 87% of children had records for BCG vaccine, and 79% for the hepatitis B vaccine. Parents of two of the children that died reported that their children had not received any vaccinations at birth.

Eleven children (18.3%) reported having had pneumonia previously; however, none of them reported having had other known risk factors.

### Semi-structured interview results

#### Caregivers’ characteristics

Out of 63 parents/caregivers, three accepted to be interviewed during hospitalization because they lived outside Iquitos city, and 19 accepted to be interviewed after discharge. We lost contact with three of them and we could not find addresses of nine families; so we did only seven additional interviews, ten in total. We interviewed ten parents/caregivers: eight mothers and two fathers, despite the fact that the mother was identified as the main caregiver in the two cases where fathers were interviewed. We spoke to the fathers in these last two cases because the families were from rural communities and the women either did not speak Spanish (in one case) or the father told us that he would be the respondent (in another case). Caregiver’s ages ranged from 18 to 63. Three interviewees came from rural communities. Socioeconomic characteristics are shown in Table 3. These rural communities are characterized as being only accessible by boat, or in one of the cases, by small plane. Six came from urban and peri-urban areas. People living in peri-urban areas can access the urban region city by car or motorcar (rickshaw), but the roads are usually unpaved and the house materials are very rustic.

**Table 3:** Socio-economic characteristics of caregivers (*n* = 10)

Characteristics	*N* (%)
Age, in months (median (IQR))^a^	28 (24–36)
Female	10 (100%)
Level of education of the caregiver/mother	
None	0
Primary	2 (20%)
Incomplete Secondary	4 (40%)
Secondary	3 (30%)
Superior	1 (10%)
Piped water	7 (70%)
Floor material of the house	
Earth	3 (30%)
Wood	6 (60%)
Cement	1 (10%)
Number of rooms per house (median (IQR))*	2 (1–4)

^a^(median (IQR)): In these rows, values shown under (*n*) are median values and values shown under the % column are interquartile ranges

#### Delays

Data was organized using the structure from the three delays model. Quotes from key informants are presented to reinforce key messages. Applying the structure of the three-delays model to our data, we made the following observations.

#### Delay in *decision* to seek health care services

The median time to take children to a health center was 3 days. Seven caregivers reported delays of 3 to 14 days to reach a health center. Given that there were no delays in actually identifying and reaching a health facility (see later), we assume this is the time that caregivers took to decide to seek formal health care service.

There were four main factors identified as barriers to making a prompt decision to seek care: 1) lack of knowledge of the signs and symptoms, 2) reliance on self-medication, 3) lack of confidence in the health care system, and 4) need for input from other family members to make a final decision.

The first factor that emerged was an apparent ***lack of knowledge of the signs and symptoms*** of pneumonia among caregivers:

One caregiver mentioned that her child had been sick for one week before deciding to seek care at the primary health facility. When asked why she did not take her child to the health facility earlier, she answered: “*Because I thought it was just a simple fever, then when the fever was higher and the child was trembling, I took him [to the health post].”*

Some caregivers who mentioned having had previous experiences with respiratory diseases affecting their children ***practiced self-medication*** which delayed their decision to seek care. Two caregivers mentioned the medications they had used before. The first caregiver stated: “*Since I already know about this, in my house we always have salbutamol and the aerochamber.”* Similarly, the second caregiver stated: *“When he had a dry cough, I gave him dexacort (oral steroid) that the doctor had prescribed previously. I followed the same dose the doctor gave me before: at noon and at 6 pm. I already had his treatment.”* Another caregiver described treating their feverish child at home for about a week before seeking care: *“[My child] had a fever, for about a week, and I was giving him paracetamol [acetaminophen]. [He] had fever, diarrhea, and his chest ‘looked stretched’. I thought it was bronchial. I was bathing him in malva [local herb], and giving him malva, antalgina [metamizol], and paracetamol.”*

***Lack of confidence in the health*** **system** was reported as another barrier in their decision to seek care. One caregiver mentioned *“we have decided not to go to health centers anymore because most times they do not offer an adequate care for children. Thus, I tell my wife we have to go to the hospital directly, to the emergency room, so that a specialist doctor can assess our baby and give us the results. Based on this, they tell us if the baby is okay or if he needs treatment”.*

A fourth factor that emerged in two cases was the **apparent influence of other members of the family** (husbands or mothers in law) on the mother’s decision to seek care. As one mother mentioned *“...I told my mother-in-law that [my baby] had a flu and cough. One Friday… I told my partner to take him to the [charity] center... I was breast-feeding and his face looked strange. I didn’t want to call my mother-in-law because I didn’t want to scare her... I know what pneumonia is like but my husband told his mother and my mother-in-law came to see him and she told me that the baby was dying. I took him to the health post.”*

The caregiver’s husband of another child told us: “[Our child] *had a cough and fever, I told [her] to take him to the health post because I was going to work early, at 5 in the morning… I sent him to the health post again, I accompanied him in the night and he was given another injection for his fever and I took him home. In the early morning he had a fever again and I told [her] to take him to the health post at 7 in the morning*”.

Finally, sociodemographic background factors related to the caregivers are important to consider. There were two cases in which the caregivers made a prompt decision to seek care, within one day; two caregivers had both completed high school, lived very close to the HRL and one was older than the median, although this was the caregiver of the child that died due to pneumonia.

#### Delay in identifying and reaching a health care service

There were no apparent delays in identifying and reaching a health facility (either primary, secondary or tertiary) reported by the caregivers in urban and peri-urban areas: these individuals could reach a local health care facility within 15 min. The three cases in rural communities also revealed ease in reaching a primary health care facility, which, in theory, should have been able to manage their cases. However, at some critical point, the children were referred to the regional hospital (HRL). Although transportation to the HRL was provided for free, for two of the rural cases the transport took slightly over an hour. In one of the rural cases, transportation via a small plane was provided by the father’s employer.

#### Delay in receiving adequate health care service

After reaching a primary health center, caregivers took an additional 1 to 11 days to get to the HRL, where appropriate treatment was given. There were four main factors identified by caregivers that prevented getting adequate treatment: 1) lack of competence and/or lack of trust in the health center providers’ competence, 2) insufficient or inadequate, 3) economic constrain, and 4) lack of availability of health providers at health centers.

***A suggested lack of competence of the health center providers*** is alluded to in various interviews. In some cases it seems that there is a lack of recognition of symptoms, the caregivers were initially sent home with their children, but the caregivers did not trust that the provider’s call on the case was correct, and went back to the hospital, within 1 day. As one describes: *“ A man from the health post told me my son had nothing wrong, so I took him home and gave him his medicine, but it did not help so then I took him to the hospital again. The nurse told me it was only a cough but I told her it was not a simple cough and just then, my son started to cough. His face became red and he could not breathe. It was only then that he was seen by the doctor. I told him that I didn’t only want him to be seen, [I told him] ‘I want you to see what’s wrong with my son, because my little boy can’t sleep with that cough.’”*

Another mother describes a similar scenario: *“The doctor from the health post said it was a regular cold and angrily I replied ‘What do you think, Doctor, that I would bring my child here for no real reason?’ I asked the doctor ‘what if [my child] gets worse? Who is going to be responsible? Me? No, you because you do not want to see him.’ [You say my son] ‘has to have a high fever’… How is he going to have a high fever if I have given him a pill for it. ‘Look at him. My children look like that right now too,’ the doctor replied. My husband was also very angry. We came home, gave him another pill [for the fever], and we went to the hospital the next day because my son could not sleep”*

Caregivers mentioned that the ***treatment provided by primary care physicians was insufficient or inadequate*** and reacted by seeking care again as the symptoms continued or even got worse—but many of them went straight to the hospital at this point.

One caregiver mentioned: *“ [The doctor] gave my daughter amoxicilin and paracetamol because she already had fever. They are used to giving only that medication at the health post. I gave those medications to my daughter for 2 days but they did not help because my daughter’s chest looked more and more ‘pulled’. . The doctor had told me to give my daughter that medication for 7 days but I was not going to wait so long.”*

Similarly another caregiver mentioned*: “the doctor told me it was a simple cold caused by the change of weather in Iquitos. He gave me only clorfenamin (antihistamine, for allergies) and paracetamol and I gave these medications to my son but I saw no change so I took him to the hospital the same day and stayed there.”*

Furthermore another caregiver stated *…“next day he had no fever but he still felt bad, so I took him to the emergency at the Hospital Regional and they told me my son was severely sick”.*

Similar scenarios were described for other caregivers... *“We took her to the nearest health post (30 minutes away) where she was given an injection to bring the fever down. The next day on Tuesday, she was the same [still sick] but with no fever, so I took her to the emergency at the Hospital Regional where they told me ‘Your daughter is severely ill, she will be hospitalized”*

Furthermore, a father told us that when the child was taken to the health facility, he was given an injection for his fever, and then he was sent home. But by early morning, his fever had come back and he sent his child to the health facility again-- this situation continued for two weeks.

In terms of ***financial barriers***, most of the cases had the Peruvian comprehensive health insurance (Seguro Integral de Salud: SIS) that covered medical care and medicines. In three cases, the availability to pay gave them the opportunity to reach specialized care. One caregiver mentioned: *“I told the doctor I did not have money, I came to the city to buy living goods and never thought my son would became sick”.* Another mentioned: “*I could not go to the hospital that day because I did not have money to buy the medicines so I took my child back home”.* Finally, another caregiver mentioned*: “I came back home and had to ask my husband to give me money because I had run out of all I had”.*

Once reaching the tertiary health facility (HRL), some ***caregivers reported the doctors were not available***. One caregiver mentioned: “*In the hospital, it was also difficult to get attention, there was nobody who could take care of my child, we were sent from one place to another many times”.*

## Discussion

In this study we present information about the caregivers’ perceptions and actions regarding pneumonia management and their interactions with the health system in the Amazon region of Loreto, Peru. We also present clinical findings reported about 61 children seen for pneumonia during the nine months of this study. Applying the “three-delays model” [21] to explore delays for prompt pneumonia treatment, two main delays were identified: delay in *deciding* to seek care and delay in receiving *adequate* health care. These delays involved caregivers and health providers.

We identified delays in the decision to seek care: seven caregivers (of the 10 interviewed) took 3 to 14 days from onset of initial symptoms to bring their children to the closest health care facility. Previous studies have reported delay times in decision to seek care of 2 or 3 days [11, 17]. One factor associated to the delays we found was a lack of awareness about the signs and symptoms of severity of pneumonia among some caregivers. Due to the increase of child mortality during winter season in Peru, especially in the highlands, the Peruvian government carries out health promotion campaigns to increase identification of signs and symptoms of pneumonia in media. Galvez reported in 2002 that more than 80% of mothers in Lima (the capital city of Peru) knew the symptoms of pneumonia [25], but this may not be the case in Loreto -- one of the poorest regions in Peru and with higher levels of child mortality due to pneumonia than Lima. However, fast breathing seems to be one of the signs of emergency that is identified – all caregivers that arrived to the HRL gave that reason at admission. None of the caregivers mentioned chest retractions at admission – a similar finding to a study in Guatemala, where the researchers found that retraction was not recognized as a symptom [26]. In Loreto and the Amazon region in general, febrile diseases are prevalent, such as dengue and malaria [27–31]; therefore these other infections may mislead caregivers as well as primary health providers. In Kenya, children with febrile signs were less likely to be perceived as severe cases [32]. Likewise, pneumonia severity is often not recognized, likely due to the lack of awareness of pneumonia signs and symptoms [26, 32, 33].

Another factor that prevented prompt decision to seek care is that most caregivers gave some treatment at home, including herbal or more traditional medicines, which is very common in rural settings [16, 18, 34]. Several parents reported “self-medication” of their children with over the counter and prescribed medications (both medications “left over” from the past or buying new medications) from previous experiences with similar symptoms [18, 32]. Purchasing prescribed medications without an updated prescription or any prescriptions) is a common practice in Peru, since most medicines can be easily bought without prescriptions [35].

The lack of confidence and trust in the primary health personnel also resulted in delays in seeking treatment. This is a common situation in low and middle income countries. Another study in the Peruvian highlands [20], as well as in Africa [32, 36], found that many people do not trust primary health personnel, mainly because of negative staff attitudes, limited hours, lack of skilled medical personnel or lack of treatment drugs. Prior negative experiences led to people losing trust in their health services, and prevented some people from seeking care in health centers.

We noticed some degree of dependence on third persons, such as the mother in law or the husband. It is well known that in indigenous communities in the Peruvian Amazon, the male role is “dominant” within the family dynamics [37]. In Guatemala, results from a national-based survey revealed that mothers sought advice from relatives [33]; there is no information about the reason for this, but there may be issues of depending on others for decision making. This has also been reported as a factor related to delays in seeking care in Africa, especially in locations where the social interaction between women and men lead to women depending on their husbands [34, 36].

Finally, our findings indicate that parents who took their children earlier to the hospital had more education than parents who took longer. Various studies have found that maternal education is an important determinant of children’s health [15, 38].

In general, issues related to cost of treatment or lack of money did not emerge in the interviews. It seems that the SIS (the Peruvian universal insurance that covers all children under 5) is providing sufficient coverage in these cases. However, we noticed that some parents were not aware of the insurance and found out about it when they reached the health facility. This lack of awareness about the SIS has been reported previously in the Peruvian Amazon [39]. In other settings in Africa, financial constraints to cover other costs related to the treatment, including registration fees, transportation, lab tests and prescribed drugs or other supplies, were identified as barriers that prevented seeking care [32, 36].

The third delay in the “three delays model” is the delay in receiving adequate care. We identified several cases of ineffective management of CAP in children under five in the health posts and centers that delayed prompt referral and adequate treatment. Numerous parents reported that personnel in health centers did not promptly diagnose and manage their child’s pneumonia. There was also evidence of inadequate diagnostic tools: many of the children did not have oxygen saturation readings, likely the result of few oximeters available.

One limitation of this study was that the study was hospital based, so we recruited individuals who had actually made it to the hospital. There is a physician at most primary health centers in Peru, hence, we assumed this physician would be most parents’ first contact within the health system. All parents, even those living in rural settings, reported that they could reach the health facilities within 30 min -- no delay in seeking care was reported. However, because this was a hospital based study -- we do not have a registry of what happened to people that did not get to a health center, or who might have resolved their case at the health center without need of seeking care in the hospital. As stated previously, Loreto is a big region, with a dispersed and, in many cases, many villages that are isolated from one another and their capital city. There are only two main hospitals, and these are both in the capital city, Iquitos. Communication and transportation is mainly through the river, and many hours or days are needed to get to those hospitals for those in rural areas. Transportation may be an important factor associated to severe pneumonia, as reported in a study in rural Philippines [40]. In Peru, the SIS will pay for transportation to higher level health facilities, like a hospital, if one is referred to it. However, fast access to a hospital is not always feasible even then: sometimes there are no ambulances or the lack of roads affects rapid transportation [19]. Another important limitation is that, although this was mainly a qualitative study, for logistical reasons the sample was small. We were able to identify certain themes, but could not stratify the analysis of the semi-structured interviews.

In Peru, the health system structure may constitute a problem. CAP is supposed to be managed at the first level of care; however the assessment is based on a WHO algorithm, which may not be sensitive enough [41] and children be referred to a tertiary hospital even for mild cases. An alternative would be to have better diagnostic tools, that can be applied easily in primary care facilities [19].

In general, the children included in this hospital based study had similar clinical characteristics to those reported in other studies in different regions around the world. At admission, classical signs and symptoms of pneumonia were reported as reasons to take children to the hospital, and also were reported at examination at admission. The main reasons to take children to the hospital were fever, cough and difficulty breathing [2, 5, 12, 15]. Most children (75%) were younger than 18 months old. And although not conclusive nor measured objectively, most children seemed to have some level of malnutrition (reflected by the low levels of hemoglobin and general appearance description by physicians). Loreto has one of the highest levels of chronic malnutrition in Peru: 31.3% in Loreto compared to 18.1% at the national level [42].

The proper management of pneumonia includes the correct establishment of severity, and the appropriate use of antibiotics and criteria to change treatment if needed [43]. It has been reported that about 15% [44] of hospitalized children are hypoxemic, in our case 25% of children that were measured were hypoxemic, indicating severity of pneumonia in these children that arrived to the hospital. Hypoxia is an important risk factor for mortality due to pneumonia [44], therefore it has been proposed that pulse oximetry should help to efficiently manage cases of pneumonia [45]. An important observation in this hospital was that although 37/59 (62.7%) of children received oxygen, only half of children had been measured for oxygen saturation at admission, and only a quarter of them were hypoxemic (oxygen saturation of 90% or less), including two of the children that died. Pulse oximetry should be readily available even in primary health centers [46], and even though the HRL was equipped with a basic pulse-oximeter; many children were not evaluated by oximetry because of lack of availability of enough oximeters. The lack of information about oxygen saturation apparently prevented a more efficient management of pneumonia. For instance, all children that were measured and resulted in oxygen saturation higher than 90% were not given oxygen; however, half of children that were not measured were given oxygen.

## Conclusion

We found that the main delays to get health care services for pneumonia among young children are due to a lack of their caregiver’s recognition of signs and symptoms associated with pneumonia, combined with delay by health care personnel in reaching the correct diagnosis and starting appropriate treatment. Public health interventions targeting these components may be useful for reducing delays in instituting appropriate health care for children with pneumonia in similar community settings.

## Abbreviations

CAP: Community-acquired pneumonia
HRL: Hospital Regional de Loreto (Regional Hospital of Loreto)
IQR: Inter quartile range
LAR: Legal authorized representative
SIS: Seguro Integral de Salud (Peruvian comprenhesive health insurance)
SSI: Semi-structured interviews
